# Zero-profile implant (Zero-p) versus plate cage benezech implant (PCB) in the treatment of single-level cervical spondylotic myelopathy

**DOI:** 10.1186/s12891-015-0746-4

**Published:** 2015-10-12

**Authors:** ZhiDong Wang, RuoFu Zhu, HuiLin Yang, MinJie Shen, Genlin Wang, Kangwu Chen, Minfeng Gan, Mao Li

**Affiliations:** Department of Orthopaedic Surgery, The First Affiliated Hospital of Soochow University, 188 Shizi Street, Suzhou, 215006 China

**Keywords:** Intervertebral fusion device, Spinal fusion, Efficacy, Internal fixation, Cervical spondylotic myelopathy

## Abstract

**Background:**

Anterior cervical discectomy and fusion is the golden standard for anterior surgery treating elderly cervical degenerative disease, but the previous implant has some problems such as looseness, translocation, sinking and dysphagia, So Zero-p implant and PCB implant have been developed to decrease the complications.

**Methods:**

The clinical data of 57 patients with single level cervical spondylotic myelopathy were retrospectively analyzed. 27 patients adopting Zero-p interbody fusion cage as implant (Zero-p group) and 30 patients adopting integrated plate cage benezech (PCB) as implant (PCB group) from January 2010 to October 2012. Observe whether are differences between the two groups of patients on operation time, intraoperatve blood loss,Japanese Orthopaedic Association (JOA) scores before and after operation, intervertebral height, cervical physiological curvature, fusion rate, Postoperative dysphagia rate and complications.

**Results:**

Zero-p group’s operation time is 98.2 + 15.2 min and its intraoperatve blood loss is 88.2 + 12.9 ml, both of which are lower than those of PCB group (109.8 + 16.9 min,95.2 + 11.6 ml ), so their differences are statistically significant (*P* < 0.05). The two groups’ JOA scores 3 months after operation and in the last follow-up are significantly higher than those before operation, so the differences are statistically significant (*P* < 0.05). Coob angle 3 months after operation and in the last follow-up improves obviously compared with before operation, so the difference is statistically significant (*P* < 0.05). The two groups’ operation segments intervertebral height 3 months after operation and in the last follow-up improves obviously compared with before operation, so the difference is statistically significant (*P* < 0.05) Zero-p group has one patient with dysphagia after operation and PCB group has four patients with dysphagia after operation, so there is no statistical differences between the two groups on dysphagia rate (*P* > 0.05, *P* = 0.415). PCB group has two patients with screws backing out and two patients with hoarseness after operation, the two groups’ operation segments all saw bony union in the last follow-up. Zero-p group postoperative complications are lower than PCB group (*P* < 0.05, *P* = 0.044).

**Conclusions:**

Zero-profile implant and PCB implant both achieved good clinical effects on the treatment of cervical spondylotic myelopathy, the two groups both saw bony union in operation segments, but Zero-profile implant has the advantages of easy operation, short operation time, less intraoperatve blood loss and less complications.

## Background

Anterior cervical discectomy and fusion is the golden standard for anterior surgery treating elderly cervical degenerative disease [1]. Currently, interbody fusion cage replaces autogenous iliac to become the main way of posterior lumber intervertebral fusion, but it has some problems such as looseness, translocation, sinking and a low fusion rate of fusion cage [2, 3] which can be solved by implanting titanium plate before cervical spine, then dysphagia, screws backing out, the looseness of titanium plate and other complications after operation arise [4–8]. The integrated cage and plate device (the plate cage benezech ,PCB) (SCIENT’X, Paris, France) combines the advantages of anterior cervical plate and fusion cage to overcome the above disadvantages [9]; Zero-p implant (Synthes GmbH Switzerland, Oberdorf, Switzerland) can be contained completely by intervertebral space overcoming traditional titanium plate method’s dysphagia problem [10]. Currently, there are only a few clinical researches about Zero-p interbody fusion cage and integrated anterior cervical plate cage benezech implant (PCB) treating cervical spondylotic myelopathy abroad [10–13]. From January 2010 to October 2012, our department adopted Zero-p implant to cure 27 patients with cervical spondylotic myelopathy and PCB implant to cure 30 patients with cervical spondylotic myelopathy and compared the clinical effects of the two methods.

## Methods

**Fig. 1:**
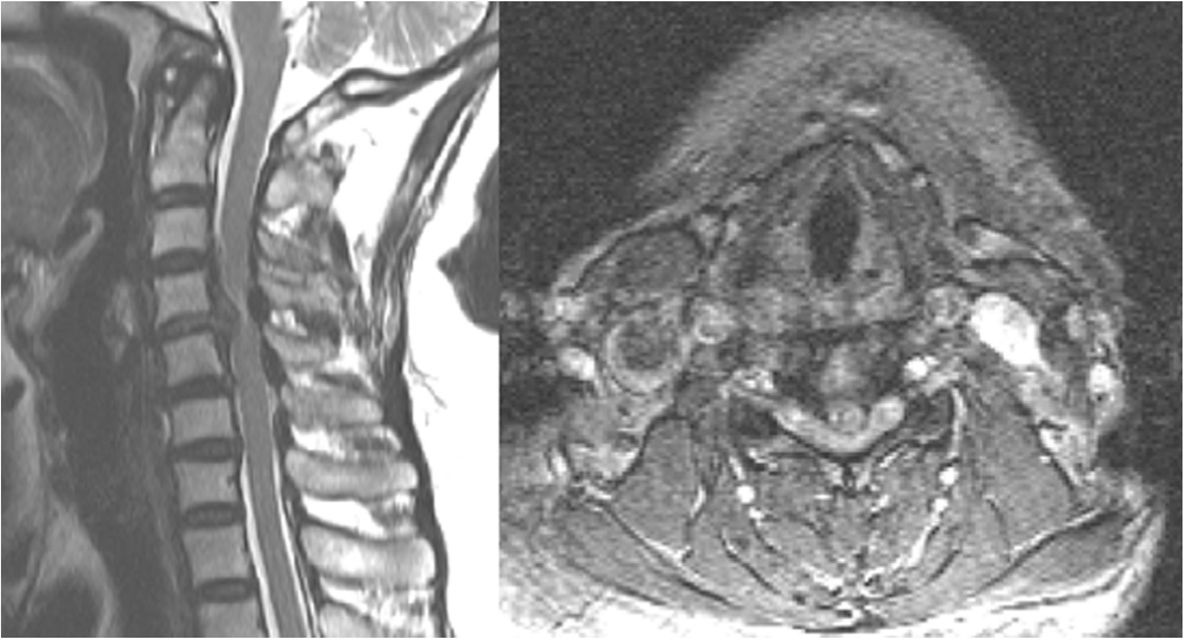
Sagital T2 magnetic resonance image of a typical study patient showing single-level cervical spondylotic myelopathy (C4-5)

The inclusion criteria: 1. The patients have the typical symptoms and signs of cervical spondylotic myelopathy and formal conservative treatment is not effective; 2. The patients were diagnosed with single level cervical spondylotic myelopathy by CT or MRI (Fig. 1); 3. The patients have constant and complete clinical and image materials;

The exclusion criteria: 1. The main symptoms are axial symptoms and root symptoms rather than myeloid symptoms; 2. the patients have cervical vertebra surgery and other cervical vertebra disease records including fracture, tumor, ossification of posterior longitudinal ligament and so on; 3. Anterior operation and posterior operation were conducted simultaneously.

Fifty seven patients with cervical spondylotic myelopathy conforming to inclusion and exclusion criteria were recruited by the research from January 2010 to October 2012 which were divided into two groups according to the implants used in ACDF: 27 patients adopted Zero-profile impalnt (Zero-p group) and 30 patients adopt PCB implant (PCB group). Refer to Table 1 for the detailed materials of the two groups of patients which are comparable. All patients had written informed consent for participation in the study. This study was approved by the Institutional Ethics Committee of Soochow University.

**Table 1:** Preoperative patient data and operated levels in the two groups

Group	Age (years)	Sex (male/female)		Operated level			
		Male	Female	C3-4	C4-5	C5-6	C6-7
Zero-p group	51.6 ± 11.3	12	15	2	8	9	8
PCB group	54.0 ± 8.5	12	18	3	7	12	8
Statistical value	*T* = 0.924	*χ* ^*2*^ = 0.115		*χ* ^*2*^ = 0.540			
P value	0.36	0.734		0.910			

## Surgical procedure

**Fig. 2:**
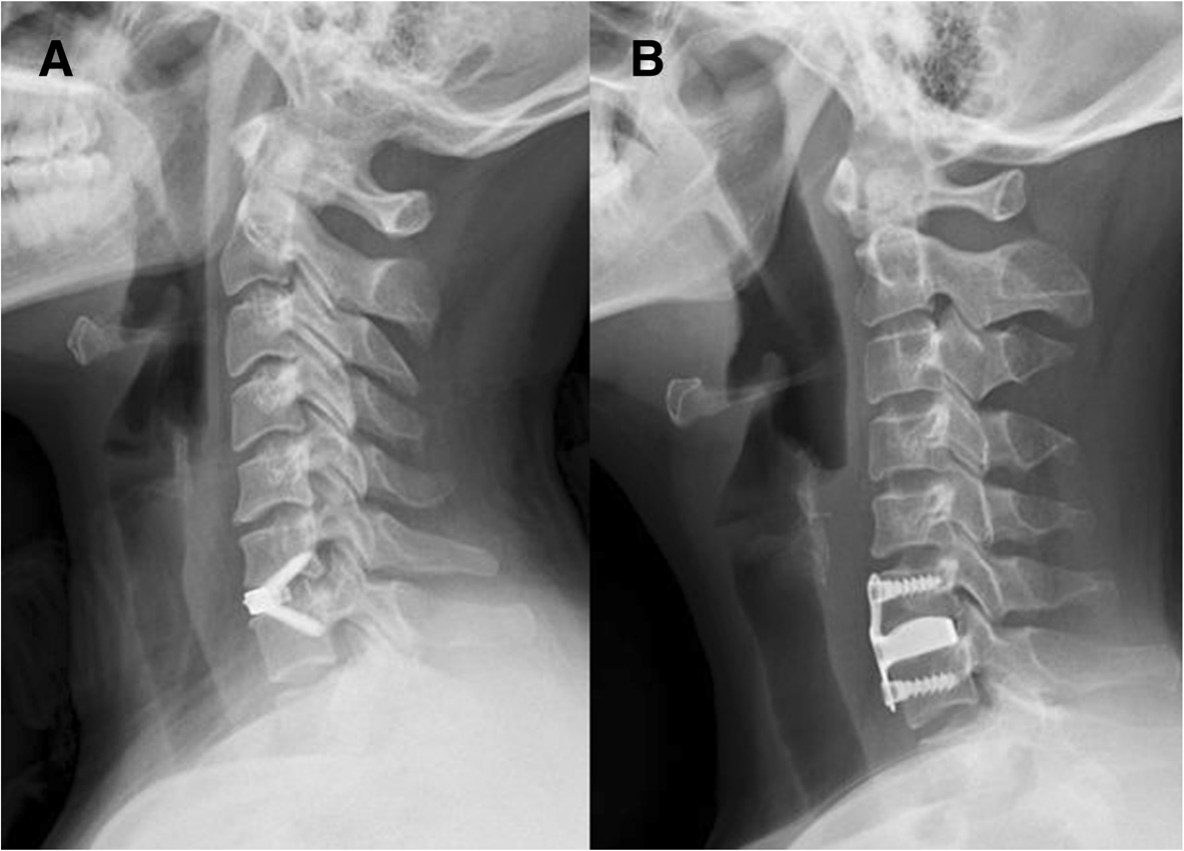
Postoperative lateral radiographs showing **a** a patient with C6–7 anterior cervical discectomy and fusion (ACDF) with a Zero-P implant, and **b** a patient with C6–7 ACDF with a PCB implant

All the surgeries were carried out by a chief physician of our hospital. After successful endotracheal intubation for general anaesthesia or cervical plexus anesthesia, the surgical procedure was performed using a standard anterior cervical discetomy and fusion (Smith–Robinson approach). After confirmation and exposure of the appropriate vertebral levels, a discectomy was performed and a highspeed burr was used to remove the cartilaginous end plates from the adjoining vertebral bodies in order to prepare for bone grafting; excessive removal of the subchondral bone was avoided. The posterior longitudinal ligament, osteophytes, and other compressive elements were also removed. After testing the intervertebral height and width, the appropriate Zero-P implant or PCB implant filled with bone chips (harvested from the iliac crest) was implanted into the prepared intervertebral space. After removal of the Caspar distracter, the self-tapping screws were used cranially and caudally to fix the Zero-P implant and PCB implant. After the operation, a collar was not used. Representative lateral radiographs after ACDF with Zero-P implant and plate cage benezech (PCB) implant are shown in Fig. 2

### Clinical outcome assessment

Record operation time, intraoperatve blood loss and the occurrence rate of complications and dysphagia in and after operation with intraoperative complications including spinal cord injury, vertebral artery injury, esophageal injury, superior laryngeal nerve and recurrent laryngeal nerve injury, and postoperative complications including the looseness and pulling away of screws, the degeneration of adjacent segment, the formation of pseudarthrosis and so on. Incidence of dysphagia-related symptoms was recorded using the system defined by Bazaz [7]. Neurological function evaluation adopts 17 scoring standards made by Japanese Orthopaedic Association (JOA) [14].

### Radiological evaluation

Conduct regularly anteroposterior and lateral film examination of cervical vertebra 1 and 3 months after operation and in the last follow-up, selectively conduct CT and MRI examination and evaluate cervical physiological curvature, the recovery of intervertebral height [15] and operation segment fusion. Fusion criteria is evaluated comprehensively according to X-ray film combining CT sagittal reconstruction [16, 17]. Fusion criteria include: 1. bone trabecula passes through fusion cage and the interface of centrum; 2. There is no transparent belt between fusion cage and the interfaces of upper and lower centrum; 3. CT sagittal plane shows that continuous bone trabecula passes through the gap between interbody fusion cage and adjacent end plate.

### Statistical analysis

Adopt SPSS 17.0 statistical software to analyze data and the obtained data is expressed by $\overline{x}\pm s$. Conduct sample t test to the paired data, conduct the independent sample t test to the non-paired data and use chi-squared test to test the classified variables. The two-tailed test results were considered significant when p was less than 0.05. All the analyses were performed using Microsoft Excel 2003 (Microsoft, Seattle, WA, USA) and the Statistical Package for the Social Sciences (SPSS, Chicago, IL, USA).

## Results

All patients were fellow up with Zero-p group fellow up for 24–48 months averaging 35.2 months and PCB group fellow up for 24–49 months averaging 35.5 months. There is no statistical difference on the follow-up time of the two groups (*P* > 0.05). The operation time and intraoperatve blood loss of Zero-p group are both lower than those of PCB group. The difference between the two groups is significant statistically (*P* < 0.05) (Table 2).

**Table 2:** Comparisons of intraoperative blood loss and operative time between two groups

	Intraoperative blood loss	Operative time
Zero-p group	88.2 ± 12.9	98.2 ± 15.2
PCB group	95.2 ± 11.6	109.8 ± 16.9
T value	2.156	2.272
*P* value	0.035	0.009

Refer to Table 3 for JOA scores at different time points (before operation, 3 months after operation, the last follow-up), the JOA scores inside group 3 months after operation and in the last follow-up are all higher than those before operation, and the difference on that is statistically significant (*P* < 0.05). There is no statistical significance on the difference between the two groups on JOA scores at the same time points (*P* > 0.05). The postoperative NDI scores (three months after operation, the last follow-up) in the two groups differed significantly from their respective preoperative NDI scores (p < 0.05). There is no statistical significance on the difference between the two groups on NDI scores at the same time points (P>0.05) (Table 4).

**Table 3:** Comparisons of Japanese Orthopaedic Association (JOA) scores between two groups

Group	Case	Preoperative JOA score	3 months Postoperative JOA score	Final follow-up JOA score
Zero-p group	27	8.9 ± 1.1^**^	14.1 ± 1.5^*,**^	14.3 ± 1.5^*,**^
PCB group	30	8.7 ± 1.4	13.9 ± 1.5^*^	14.1 ± 1.6^*^

^*^Compared with the same group of preoperative *P* < 0.05; ^**^compared with PCB group at the same time points *P* > 0.05

There is statistical significance on the difference on the recovery of intervertebral space height and cervical physiological curvature (Cobb angle) of the two groups’ patients 3 months after operation and in the last follow-up. The intervertebral space height and cervical physiological curvature of the two groups of patients in the last follow-up are lower than those 3 months after operation, but there is no statistical significance on that difference (Table 5). The two groups’ patients all saw their bony union on operation segments in the last follow-up, there is no statistical significance on that difference.

**Table 4:** Comparisons of Neck Disability Index (NDI) scores between two groups

Group	Case	Preoperative NDI score	3 months Postoperative NDI score	Final follow-up NDI score
Zero-p group	27	24.2 ± 4.1^**^	14.1 ± 2.2^*,**^	13.8 ± 1.9^*,**^
PCB group	30	24.9 ± 3.7	14.4 ± 2.3^*^	14.1 ± 1.8^*^

^*^Compared with the same group of preoperative *P* < 0.05; ^**^compared with PCB group at the same time points *P* > 0.05

**Fig. 3:**
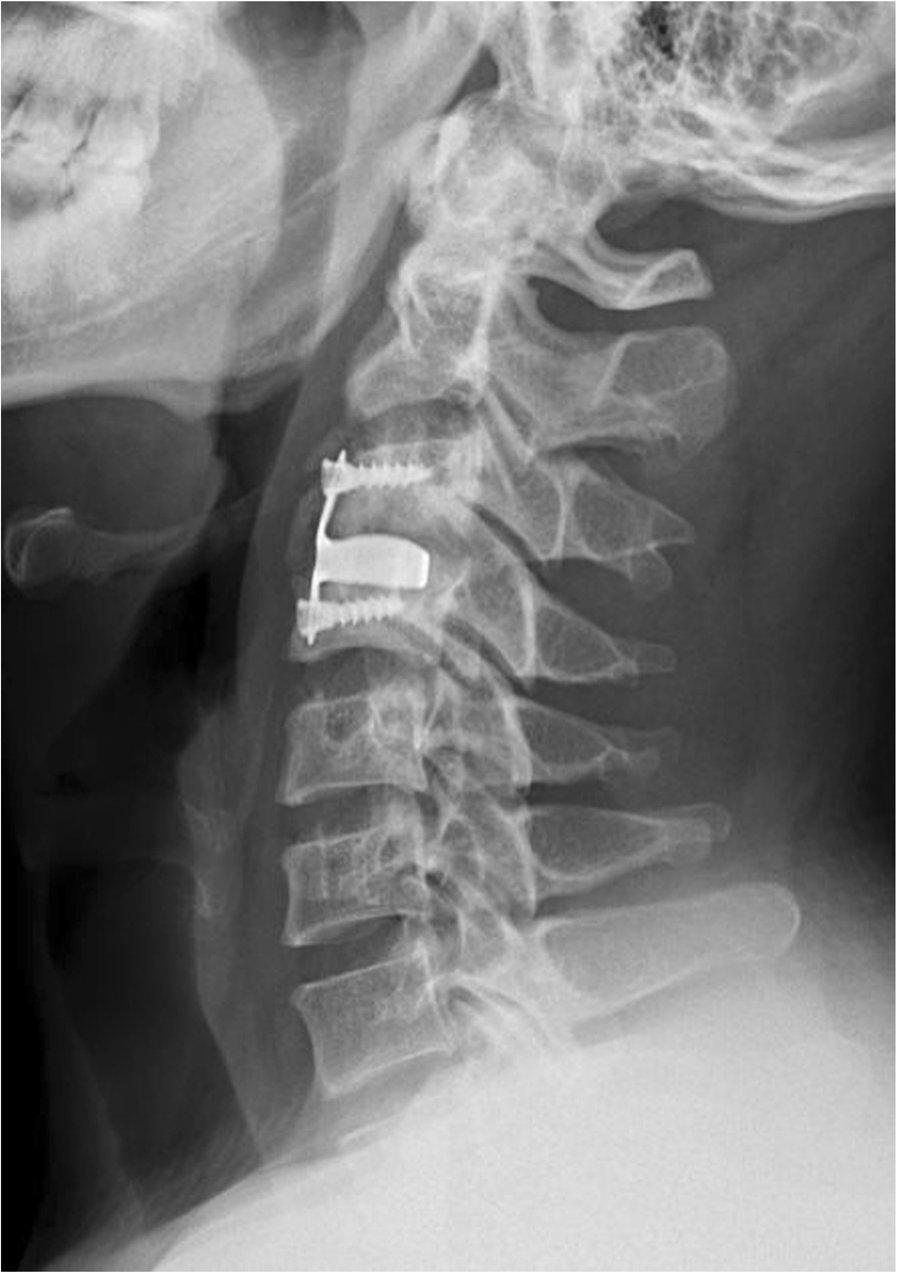
Lateral radiograph showing C3 and C4 screw loosening and heterotopic ossification formed 5 months after the operation

The operations for the two groups of patients are successful without damaging esophagus, spinal cord, vertebral artery and superior laryngeal nerve in the operation. Zero-p group had one patient with dysphagia 3 days after operation, which disappeared after 3 months’ conservative treatment, with the dysphagia rate of 3.7 % (1/27);the patient with dysphagia had recovered by self-healing in zero-p group. PCB group had two patients with dysphagia 3 days after operation and two patients with dysphagia one week after operation, which of three patients disappeared 3 months after operation and which of one patient still existed in the last follow-up, with the dysphagia rate of 13.3 % (4/30); the three patients with dysphagia had recovered by self-healing and one patient with dysphagia remain unchanged in the last follow-up in PCB group. There is no statistical significance on the difference of the two groups (*P* > 0.05). PCB group has two patients with loose implant screws (6.7 %, 2/30) (Fig. 3) and two patients with hoarseness after operation (6.7 %, 2/30) (Table 6), all of whom recovered after dehydration detumescence nerve nutrition treatment one month after operation. The two group of patients don’t have chronic pain in graft area and other complications. There is statistical significance on the difference between the two groups on complications (*P* < 0.05, *P* = 0.044) (Table 6).

**Table 5:** Comparison of intervertebral height and cervical physiological curvature (Cobb angle) at different time point between 2 groups

	Intervertebral height (mm)		Cobb angle (°)	
	Zero-p group	PCB group	Zero-p group	PCB group
Preoperative	5.24 ± 0.73^*^	5.24 ± 0.66	20.63 ± 5.05^*^	20.27 ± 4.53
3 months Postoperative	8.27 ± 0.69^*,**^	8.22 ± 0.64^**^	26.44 ± 4.09^*,**^	26.67 ± 4.15^**^
Final follow-up	8.16 ± 0.68^*,**^	8.13 ± 0.66^**^	25.96 ± 3.98^*,**^	26.23 ± 4.07^**^

^*^compared with PCB group at the same time points *P* > 0.05 ^**^compared with the same group of preoperative *P* < 0.05

**Table 6:** Complications Encountered With Each Procedure Type (Number of Patients)

Zero-p group (27 patients)	PCB group (30 patients)
Dysphagia (1)	Dysphagia (4)
	Screw back out (2)
	Temporary dysphonia (2)

## Discussion

Many technical reports appear since Cloward et al. [18] reported the feasibility of anterior decompression intervertebral bone graft fusion treating degenerative cervical disease in 1985. If bone graft or intervertebral fusion cage fusion operation is completely depended on, the long-term neck collar fixation is needed and the collapsing and pulling away of bone graft, the looseness and translocation of fusion cage, a low fusion rate and other complications are likely to appear. The use of anterior titanium plate not only makes up the disadvantages of the single use of bone graft or interbody fusion cage but can provide immediate stability and improve intervertebral bone graft fusion rate. Though the use of titanium plate reduces the occurrence rate of complications compared with the single use of bone graft or interbody fusion cage, it leads to the occurrence of the looseness, pulling away and fracture of screws, dysphagia and other complications. Zero-p interbody fusion cage in the research adopts zero incisure concept in its design, it can be contained completely in intervertebral space after being implanted, so the interference on prevertebral soft tissue and esophagus is reduced. In biomechanical stability, Scholz et al. [19] found through research that there is no difference between Zero-p implant and the fixation of titanium plate along with cage. Integrated anterior cervical plate cage benezech implant (PCB) was invented by French Benezech having the advantages of anterior cervical titanium plate and interbody fusion cage and overcoming the disadvantages of sliding and looseness of bone graft . Samandouras [9] and other researchers’biomechanical experience shows that the position changing value of PCB in the state of compression and stretching is lower than the photodynamic action of healthy spine under 50 N. The results of the research show that the two used implant methods both can maintain operation segments intervertebral height and cervical physiological curve. Integrated plate cage benezech is made of titanium alloy, which doesn’t influence magnetic resonance imaging but influences the imaging evaluation of bone fusion because X-ray can’t penetrate it; three-dimension CT examination increases patients’ economic burdens.

PCB is an integrative structure of interbody fusion cage and steel plate including an integrated steel plate and a hollow interbody fusion cage. It has the superiority of fusion cage and the safety of steel plate and the shape of convex in the top and straightness at the bottom of fusion cage conforms to the anatomy features of intervertebral space and it can clamp automatically. The fixation steel plate at the end inclines backward 10°, which conforms to the physiological property of lordosis and is longer than the steel plate at the end so that steel plate can closely adhere to the front surface of centrum at the top and the system can be reinforced on the three points, equaling to providing a good postoperative stability. The common complication after anterior cervical decompression and fusion is dysphagia. Bazaz et al. [7] reported the dysphagia rate increased after anterior surgery use titanium plate fixation. After comparing anterior titanium plates with different thickness, Lee [20] and other researchers think there is a direct relation between the thickness and texture of titanium plate and dysphagia that the more smooth and thinner titanium plate is and the smaller the stimulation of titanium plate to prevertebral soft tissue and esophagus is, the lower the occurrence of dysphagia after operation is. Tortolani et al. [8] reported that the early dysphagia rate after anterior cervical decompression and fusion is 2–67 %. Literature [8, 20] reports that the dysphagia rate 3 months after anterior cervical surgery using titanium plate is 12–35 %, Koller et al. [21] reported that after anterior cervical decompression and fusion, 17.6 % patients had transient dysphagia. In the research, PCB group had an early dysphagia rate of 13.3 % conforming to the reported dysphagia rate. Zero-p has a lower dysphagia rate than that reported in literature maybe because Zero-p interbody fusion cage is completely contained in intervertebral space after decompression which reduces the separation of prevertebral soft tissues so that the stimulation of implant to prevertebral soft tissues and esophagus can be avoided.

Postoperative complications of the two group are related to intraoperative operating and implant, postoperative dysphagia of Zero-p group is related to pulling prevertebral soft tissue in operation, the early dysphagia of PCB group after operation is related to the over stretch of neighboring soft tissues in operation or the mechanical stimulation of prevertebral plate cage benezech implant to esophagus and neighboring soft tissues, the existence of dysphagia in one patient observed in the last follow-up may be related to plate cage benezech implant taking up some anterior physical space or titanium plate rubbing against esophagus or adhering to neighboring soft tissues [4, 22, 23], we can clearly seen heterotopic ossification and screw loosening on lateral view in Fig. 3. The patient with dysphagia remain unchanged in the last follow-up. The heterotopic ossification formed may be related to operative levels instability in the early period. the looseness of implant in two patients of PCB group after operation may be caused by the unlocked screws of PCB. The hoarseness in PCB group after operation may be caused by recurrent laryngeal nerve injury caused by the over stretch of prevertebral soft tissue in order to place plate cage benezech implant well in operation. Apfelbaum et al. [24] reported that the dysphonia after anterior cervical surgery is partly caused by recurrent laryngeal nerve injury in the process of trachea cannula.

Although this study demonstrates satisfactory results about Zero-p implant and PCB implant in the treatment of CSM, some limitations were presented in it, including retrospective analysis of the data and a small sample size. In addition, the surgical procedure was chosen by the patients. A larger sample size and randomized controlled trial are needed to perform.

## Conclusions

In the research, it is observed in the last follow-up that the Zero-P implant and PCB implant are effective treatments for single level spondylotic myelopathy. but the zero-p implant is easy to operate and leads less complications.
